# Modification of Fatty Acid Profile and Oil Contents Using Gene Editing in Oilseed Crops for a Changing Climate

**DOI:** 10.1080/21645698.2023.2243041

**Published:** 2023-08-08

**Authors:** Saeed Rauf, Seerat Fatima, Rodomiro Ortiz

**Affiliations:** a Department of Plant Breeding & Genetics, College of Agriculture, University of Sargodha, Sargodha, Pakistan; b Department of Plant Breeding, Swedish University of Agricultural Sciences, Alnarp, Sweden

**Keywords:** Gene editing, genome, induced mutation, oil contents, sunflower

## Abstract

Mutation breeding based on various chemical and physical mutagens induces and disrupts non-target loci. Hence, large populations were required for visual screening, but desired plants were rare and it was a further laborious task to identify desirable mutants. Generated mutant had high defect due to non-targeted mutation, with poor agronomic performance. Mutation techniques were augmented by targeted induced local lesions in genome (TILLING) facilitating the selection of desirable germplasm. On the other hand, gene editing through CRISPR/Cas9 allows knocking down genes for site-directed mutation. This handy technique has been exploited for the modification of fatty acid profile. High oleic acid genetic stocks were obtained in a broad range of crops. Moreover, genes involved in the accumulation of undesirable seed components such as starch, polysaccharide, and flavors were knocked down to enhance seed quality, which helps to improve oil contents and reduces the anti-nutritional component.

## Introduction

Agriculture must substantially increase its productivity, lower its environmental footprints, cope with climate change, and provide renewable alternatives to fossil oil. Oilseed crops such as oil palm, soybean, sunflower and brassica species provide a cheap source of vegetable fat for human diets. Increasing the oil contents and modifying the fatty acids profile of oil seed crops is an important breeding objective, thus enabling crops to expand their industrial applications and utilization in numerous processes.^1^ Generally edible oil of various oilseed crops such as soybean or sunflower has been modified to improve their cooking quality by reducing their rancidity and oxidation helping them to tolerate high temperature and improved shelf life of edible oil.^2^

Climate change is a major challenge for the sustainable production of oilseed crops. It is the result of the human activity that led to rapid use of fossil fuels, industrial effluents and greenhouse gas emissions. Effects of the climate change are more obvious in terms of heat waves, drought, flood, salinization and effluents contaminating the soil. Rapid greenhouse gas emissions have raised atmospheric temperature and influenced crop water demand by various oilseed crops. Moreover, salinity stress impact has also been increased many fold due to cumulative effect of high temperature and drought. These abiotic stress induced production of harmful reactive oxygen species which caused cell membrane leakage, deterioration of proteins structure and function, toxic effect of harmful ions. The cellular impact of stresses may also induce downregulation of transcripts involved in the cellular metabolism.^3^

New plant breeding tools have been developed that can significantly contribute to improving oil crops. They include different genetic enhancement methods for the incorporation and identification of novel alleles related to the fatty acids and oil contents.^4^ In the past, recurrent selection cycles were pursued to increase oil contents of sunflower. Pustvoit used the method of seed reserves (modified recurrent selection) for improving this crop, which increased its oil content from 30 to 40% after a decade of selection cycles (1934–1944).^5^ Baldur Stefenson and Keith Downy exploited genetic diversity in brassica germplasm to develop rapeseed (renamed as “canola” in North America) versions of *Brassica napus*, and *Brassica campestris*, which had very low concentration of erucic acid (22:1).^6^ These canola cultivars were accepted by growers and expanded on large scale for cultivation because erucic acid has been regarded as potentially dangerous for cardiovascular health.

**Figure 1.**
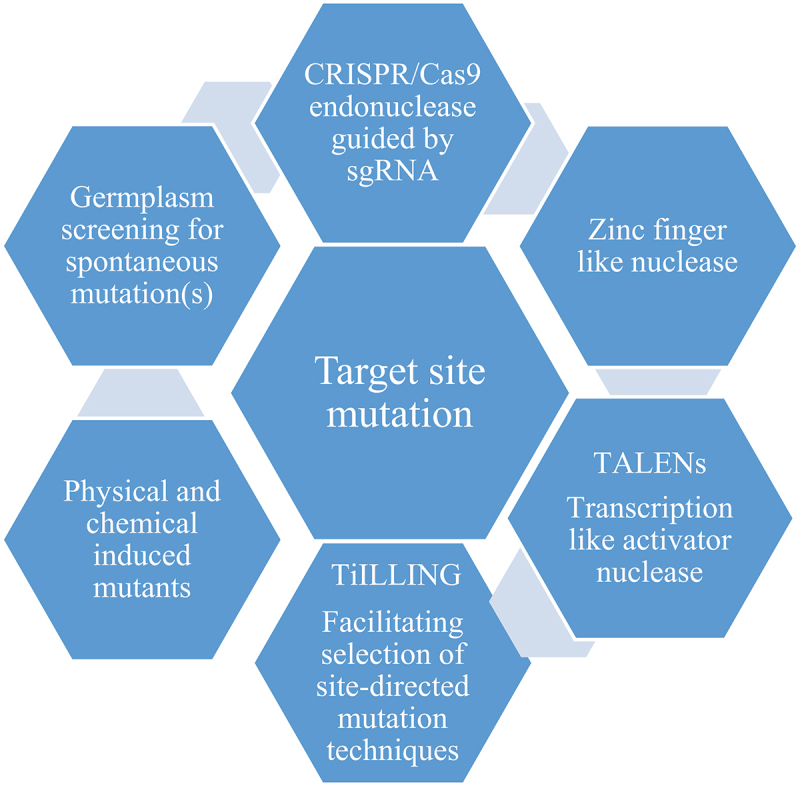
Optional methods for inducing mutation in the desired gene or traits.

Options to induce random or site direct mutations are shown in Fig. 1. Chemical and physical mutagens were exploited to induce mutations in genes such as *fatty acid desaturase 2* (*FAD2)* for modification of fatty acids in sunflower and soybeans. Several other techniques such as “TILLING” were devised to induce site directed mutation for targeted loci.^7^ These techniques are, however, laborious and time consuming to identify desirable mutant in large populations. Furthermore, novel genetic engineering tools were exploited such as antisense RNA technology to silence undesirable genes that lower edible oil quality or RNAi to control RNA translation.^8^ Nevertheless, a drawback of genetic engineering-derived products is to pass hurdles and legislation required for releasing them for farming, and the lack of adoption due to concerns in the population over the use of transgenic crops carrying foreign genes and markers.^9^

**Figure 2.**
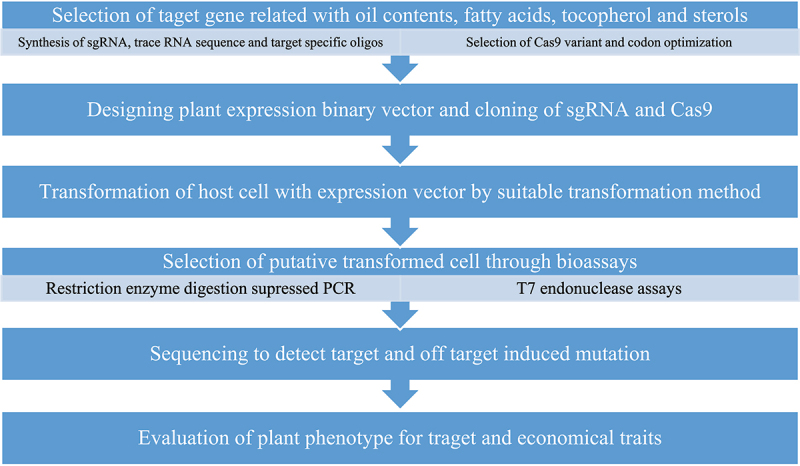
Steps for development of genetic stocks through CRISPR/Cas9-mediated target gene mutation.

The Clustered Regularly Interspaced Short Palindromic Repeats/Cas9 (CRISPR/Cas9) can induce site directed mutation by downregulating the targeted genes.^10^ This may help to silence undesirable genes. However, CRISP/Cas9 may also help to upregulate the targeted genes by incorporating few nucleosides in the frame.^11^ It has been found that chances of mutant stock to reach an operational technology readiness level success was greater, as they are free from foreign contamination and marker genes and free from undesirable effects of mutation at non-target loci.^9,12^ Figure 2 shows the process for gene editing using CRISP/Cas9-mediated target gene mutation.

Cooking and edible quality of oils is dependent on specific concentration of fatty acids, flavonoids, tocopeherols and sterols.^13^ For instance, high oleic or stearic acid edible oils were preferred for cooking and frying purpose over high linoleic acid, which show oxidation and rancidity during cooking.^13^ Similarly, omega 3 fatty acids were required to increase edible value, industrial manufacturing of paints, varnishes and nutraceuticals.^13^

### Genomic Diversity Among Polyploid Species

There are numerous oilseed crops with diversified origin and genome evolution. These species carried single or multiple copies of the genome; i.e. sunflower or olives have a single copy of genome, while soybean, or rapeseed (*B. napus*, *B.juncea*), possesses more than one copy of the ancestral genome with allelic redundancy for single loci, thus making CRISPR/Cas9-mediated gene editing complicated to inactivate multiple homologues for single genes.^14^ In tetraploid or hexaploidy plants, three or four homologues should be inactivated, which led to the development of monoallelic or biallelic mutants in various oilseed crops such as soybean.

### Off Targets Effect of Multiple Allelic Knock Out on Plant Phenotype

Every new technology has some limitations; i.e., off target effects on plant phenotypes or pleiotropic effects related with specific gene inactivation could be one of the problems related with gene editing using CRISPR/Cas9. The sng RNA was designed to inactivate several homologues within the genome. However, it may induce mutation in unrelated gene(s) due to faulty design or close resemblance with the target gene(s), or it may induce mutation in a pleiotropic allele controlling other plant traits that may lead to undesirable plant phenotypes. Gene redundancy in polyploid species could also mask the mutant genotypes even when the gene knockout occurred in the target site.^15^ Double mutants were developed through crossing lines containing either of single mutant gene *bnc06.ida* and *bna07.ida* involved in floral abscission of rapeseed.^15^ DNA markers were used to discriminate between heterozygous and homozygous genotypes and finally doubled mutants were recovered in the F_2_ population through speed breeding within six months.^15^ Several protocols have been proposed to reduce the off target inactivation of genes, which were described with details by Naeem et al.^16^ These proposed protocols include “biased or unbiased off-target detection, cytosine or adenine base editors, prime editing, dCas9, Cas9 paired nickase, ribonucleoprotein (RNP) delivery and truncated gRNAs.”^16^

### Correction Morpho-Phenological Defects in Oilseed Crops

Some oilseed crops such as brassicas and sunflower have defects such as shattering and nonuniform ripening, disease susceptibility, and antinutritional seed component. Moreover, modification in the plant architecture may also help to improve the yield components. Number of siliques plant^−1^ were increased as result of mutation in plant architecture genes; i.e., *CLV1* or *CLV3*.^17^ Lines bearing the mutation in genes *CLV1*, *CLV2* and *CLV3* mediated by NHEJ-CRISPR/Cas9 were developed in *Brassica napus*.^18^ The line bearing mutation for *BnCLV3* was more successful due to stability in the expression of yield components; i.e., number of seeds silique^−1^, seed weight and the number of leaves per plant than wild-type controls.^18^ Research for inducing mutation for yield components remains scanty due to partial redundant genomes in polyploidy species and existence of several loci affecting a single trait. For instance, *ap1* mutants of *B. napus* was known to affect plant architecture and was related with production of a greater number of ectopic floral buds and shoot branching.^19^ The locus *ap1* may be also compensated by *CAULIFLOWER* (*CAL*) gene in the Brassicaceae family.^19^ Mutant lines bearing multiple *SHP1* and *SHP2* were developed by targeting six *BnSHP1* and two *BnSHP2* homoeologs by the CRISPR/Cas9 genome editing system.^20^ Mutation *BnSHP1A09* was involved in controlling lignin contents at dehiscence zone.^20^ Simultaneous mutation of *BnSHP1A09/C04-B/A04* and *BnSHP2A05/C04-A* exhibited reduced lignified layer and separation layer adjacent to valves and replum. The pod-shattering resistance index (SRI) subsequently increased to 0.31 in five homoeolog mutation lines of *Brassica napus*.^20^

Soybean and sesame germplasm were adapted to specific photo-thermal conditions. The application of knockout gene technology may help to identify the gene functions related to the photo-thermal plant responses and subsequently modification of these genes to develop germplasm with novel phenological traits (Table 1). The CRISPR/Cas9 gene editing method was used to induce mutation in the *E1* locus that suppress early flowering in soybean under long daylength. A mutation was induced at two sites within the genome; i.e., 11 and 40 bp deletion, which caused early termination of translation, thus ending up the production of truncated protein.^27^ A double mutant ft2aft5a induced through CRISPR/Cas9 expressed delayed flowering by 31.3 days under short days, thereby resulting in increased pod and seed yield.^27^ It was concluded that delayed flowering may be beneficial in tropical environments.^27^ In contrast, a modification of flowering inhibiting gene *GmPRR37* through CRISPR/Cas9 induced early flowering in soybean.^28^ A natural mutant allele *Gmprr37* enabled cultivation of soybean at high altitudes and provide adaptability to specific regions.^29^

**Table 1.** CRISPR/Ca9 mutants with modified pheno-morphological traits in rapeseed and soybean.

Species (crop)	Gene	Effect	References
*Brassica napus* (rapeseed)	*BnSHP1A09/C04-B/A04* and *BnSHP2A05/C04-A*	Mutation reduced lignified layers and separation layers	^20^
	*BnaC04EPSPS*	Glyphosate tolerant with modification of amino acid	^21^
	*Pod dehiscence 1* (*PDH1*)	Improved the pod shattering resistance	^22^
	*BnITPK*	Knockout of three functional paralogs of soybean caused the phytic acid free soybean meal	^23^
	*AtCRT1a*	Knock out of the gene resulted in the decreased susceptibility to *Verticillium longisporum*	^24^
*Glycine max* (soybean)	*GmPRR37*	CRISPR/Cas9 induced early flowering under long days	^25^
	*Gmhdz4*	Increased above ground biomass and roots under water stress	^26^

### High Oil Contents Mutant Stocks

Oil contents were positively influenced by traits such as embryonic tissues, size and density of oil bodies and were negatively influenced by polysaccharides, pigments (proanthocyanidin) and starch accumulation in seed. Pigments deposited in the testa reduce the oil contents and deteriorate the quality of oil and meal. The sgRNAs were designed to edit a single or multiple genes within single or multiple genomes of a species (Table 1).

Seed specific gene inducing polysaccharides deposition were inactivated through CRISPR/Cas9 to increase oil contents in the seed. Cultivars with yellow seed were bred by knocking down both *NtAn1* and *BnTT8*, which are genes related to testa thickness, pigment deposition, and increased oil content in the seed (Table 2).

**Table 2.** Stocks generated through CRISPR/Cas9 gene editing.

Species (crop)	Gene	Effect	References
Arabidopsis thaliana (thale cress)	*NtAn1a and NtAn1b*	Yellow seeded mutants lacking proanthocyanidin had high protein and oil contents (15–18%)	^30^
Brasica napus (rapeseed)	*BnTT8*	Yellow seed mutant had high oil and protein contents with altered fatty acid profile but not affecting seed yield	^31^
	BnLPAT2/BnLPAT5	Oil contents decreased by 26% to 32% with corresponding increase of polysachride and	^32^
	BnSFAR1, BnSFAR4 and BnSFAR5	Seed oil content was increased in BnSFAR4.a double mutants by 12.1% and 8.9% and in BnSFAR4.b double mutants by 10.3% and 8.7% when compared with wild plants	^33^
	BnaCIPK9	Seed oil content was improved by 3–5% and 1%–6% in bnacipk9 lines and gene-silenced lines. Moreover, it had higher mono-unsaturated fatty acid	^34^
Thlaspi arvense (pennycress)	*BADC1, BADC2, BADC3*, as well as in LDAP-Interacting Protein (LDIP)	Deciphering how fatty acid biosynthesis regulation differs in pennycress compared to other brassicas, and how genetic manipulations may increase seed triacylglyceride levels	^35^
Glycine max (soybean)	*LOX1, LOX2, LOX3*	Flavor and odor free soybean oil and protein by inducing double and single mutant *gmlox1, gmlox2, gmlox3*	^36^
	BnaMAX1	Knock out of four homologues resulted in semi dwarf mutant having higher silique and seed yield potential	^37^
	GmMFT	Mutant was used to determine function of *GmMFT* and its higher expression was related with greater oil contents and seed mass	^38^
Oryza sativa (rice)	*OsLip1*	Inactivation of lipases enzyme in rice bran to avoid oxidation	^39^
Camelina sativa	*CsDGAT1, CsPDAT1*	Inactivation led to shrunken seed and to the discovery of their role in enhancing oil contents	^27^
Nicotiana tabacum (tobacco)	*NtAn1*	Inactivation of gene resulted in the increase of oil contents due to yellow seed coat color	^30^
Lepidium campestre (field cress)	*FAE1*, *FAD2*, *ROD1*	Mutated lines with reduced C22:1, but an increase in C18:1	^40^

Rice bran may also be a potential source of edible oil in South and East Asia. Rapid deterioration of rice bran due to oxidation is a major hindrance that reduces its utilization for large expelling of oil.^41,42^ Genetic factors inducing rapid degradation of rice bran oil have been reviewed in detail.^42^ CRISPR/Cas9 gene editing may assist to develop stocks having mutations in genes such as lipases and lipoxygenase, which cause rapid deterioration of rice bran oil. Moreover, concentration of poly unsaturated fatty acid (i.e., linoleic acid) may also be reduced to avoid oxidation and rancidity of rice bran oil.^39^

Seed oil content tends to decrease after the seed maturation and factor involved in rapid degradation may be inactivated to harvest high oil contents in seed even at maturity (Table 3). A gene known as *SEED FATTY ACID REDUCER* (*SFAR*), which is involved in the rapid degradation of oil contents after maturity in polyploid species, could keep the oil content.^43^ Inactivation of genes such as *DGAT1 and PDAT1* confirmed their role in triacyl glycerol synthesis in *Camelina sativa*.^44^ Inactivation of genes *BNlPAT2* and *BNlPAT5* and their four homologues in *B. napus* resulted in the reduction of oil contents by 32% and 29%, respectively.^32^ A double mutant for both genes had a 39% decrease in oil content.^32^ It was noted that both genes were involved in the biosynthesis of enzyme *lysophosphatidic acid acyltransferase*, which catalyzes the biosynthesis of triacylglycerols in various plant species.

**Table 3.** Low saturated fatty acid stocks generated by CRISPR/Cas9 gene editing.

Species (crop)	Gene	Effect	Ref.
Arachis hypogea (peanut)	*AhFatB*	Site directed mutagenesis of *Arahy.4E7QKU* reduced palmitic acid and increased oleic acid content	^45^
Glycine max (soybean)	*GmFATB1a* and *GmFATB1b*	Single mutant for each gene decreased palmitic acid (11%) and stearic acid (21%). while a double mutant decreased palmitic acid (35%) and stearic acid (42%)	^46^
Camelina sativa	*FAE1*	Very long chain fatty acid (20–24) by knock out three homologue of *fae1* (2%) from wild type (22%)	^47^
	FAE1	Reduced C-20 fatty acid and erucic acid and increased alpha linolenic acid and docosa-hexaenoic acid and ecosa pentaenoic acid	^47^
Brassica napus (rapeseed)	*FAE1*	Targeted mutation of 2 homologue (*BnaA08.FAE1; BnaC03.FAE1* produced 0% erucic acid (ω = 9, 22=1) with mild reduction in oil content	^48^

## Expression of Fatty Acid Genes in Vegetative Tissues and Their Relationship with Abiotic Stresses

Abiotic stress may reduce the overall fatty acid production capacity due to reduced availability of substrate and dysfunction of enzymatic machinery involved in the fatty acid biosynthesis pathways.^3^ Fatty acids such as C-18-1 (oleic acid), C-18-2 (linoleic acid) and C-18-3 (linolenic acid) are major ingredients of cellular membrane which exist in the form of phospholipids and their stable accumulation helps in coping various abiotic stress factors (e.g. cold, heat, drought, flood and salt stress). Abiotic stress induced the relative degree of unsaturation in plasma membrane of vegetative cells and tissues which increase or decrease the fluidity and also affect the membrane permeability. Higher the degree of unsaturation higher was membrane fluidity in root cells.^49^ Higher plasma membrane or total cellular unsaturation in the root cell in response to NaCl stress resulted in the increased tolerance to stress condition in species such as *B. oleracea*. Contrastingly decrease in unsaturation increased sensitivity of roots to NaCl stress in soybean. However, opposite response was obtained in *B. napus* species.

**Figure 3.**
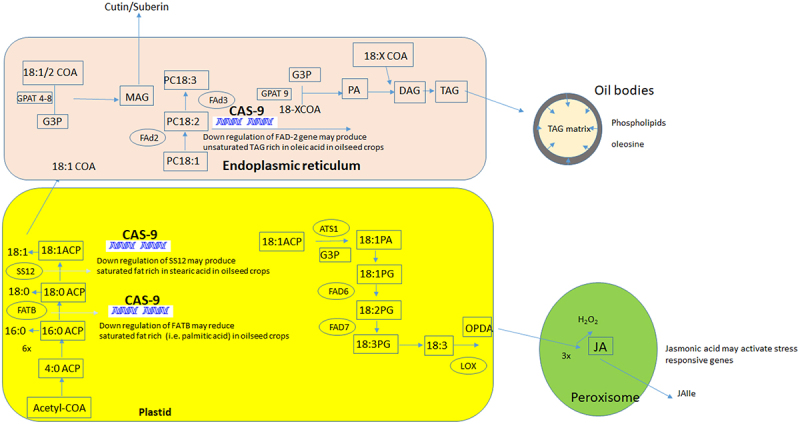
Gene editing of fatty acid biosynthesis –especially C:18 fatty acid (oleic, linoleic and linoleic acids) in two organelles; i.e., plastids and endoplasmic reticulum. The illustration explains the substrate of C18 conversion into jasmonic acid under stress in peroxisome.

ACP, acyl carrier proteins; CAS-9, clustered regularly interspaced palindromic repeat; FAD, fatty acid desaturase; FATB, acyl-ACP thioesterase B; SSI2, stearoyl-ACP desaturase; COA, coenzyme A; PC, phosphatidyl choline; MAG, monoacyl glycerol; G3P, glycerol-3-phosphate; GPAT, G3P acyltransferase; DAG, diacyl glycerol; TAG, triacyl glycerol; PA, phosphatidic acid; OPDA, 12-oxo-phytodienoic acid; PG, phosphatidylglycerol; LOX, 13-lipoxygenase; JA, jasmonic acid; JA-Ile, jasmonoyl-isoleucine.

Plant assimilate triacylglyceride (TAG) or free fatty, which may act as food reserve for embryo nourishment, could be modified by gene editing technology according to human needs. TAG or fatty acid biosynthesis in vegetative tissues was alternative source of energy upon the relieve of abiotic stresses.^49^ It was identified that cell metabolites of lipids increased under NaCl stress in rapeseed cell.^50^ Major metabolites which showed increase under stress conditions were unsaturated fatty acids related to C:18 pathways (e.g. linoleic acid, dihomo-gamma-linolenic acid, oleic acid or alpha-linolenic acid) (Fig. 3), while the metabolism of most amino acids and carbohydrates decreased. Transcriptomic followed by metabolomic pathway analysis indicated that lipid metabolism was an important response of canola roots to cope NaCl stress and osmotic stress.^50^ Fatty acid biosynthesis also provided a building block material for synthesis of waxes, cutin and suberin which may act as shield to reduce transpiration loss and insect infestation (Fig. 3).

Genes involving the elongation and unsaturation (elongase or desaturase) of fatty acids were in close proximity of the regulatory genes that activate jasmonic acid biosynthesis. A study identified 75 *FAD* genes in the soybean genome. A cis-element analysis of *FAD* promoter showed that upstream sequences of *FAD* were in close proximity of light, hormone and abiotic stress responsive *cis*-elements. Esterified and free fatty acid (C-18) oxidized to produce various oxylipins (jasmonic acid biosynthesis), which may activate stress responsive genes in relation to biotic or abiotic factors in a wide range of species including soybean.^51^ Upregulation of stress signaling pathways such as jasmonic acid may activate genes related to the reactive oxygen species. Unsaturated fatty acids (C-18) oxidized with ROS species for byproduct malondialdehyde (MDA) may be a marker of peroxidation under oxidative stress. Unsaturated fatty acids (C18:1) are also antioxidants and may consume ROS in vegetative tissues.^49^ Expression of fatty acid genes specifically in the vegetative tissue may be done in the by fusing promoters of the photosynthesis specific genes which enable to express in leaves tissues.^52^ *DIACYLGLYCEROL ACYLTRANSFERASE 2* (*DGAT2*) transcript was fused with promoter from nonspecific photosynthesis gene which increased the oil contents in leaves of *Arabidopsis thaliana*.^52^ Over expression of genes (*GmFAD3*, *FAD6*) involved in the synthesis of various kinds of desaturase enzyme resulted in the enhancement of the abiotic stress tolerance due to activation of stress signaling pathways (Table 4). On the other hand, inactivation of *FAD7* resulted in the decrease of drought and salt tolerance.^49^ Research found that over expression of *GmFAD3* gene also enhanced the expression of *GmWRK54* involved in the activation of stress signaling pathways in soybean. The increased expression of *GmWRK54* enhanced abiotic stress tolerance due to over accumulation of osmoprotectants and better cooling canopy.^53^ *GmWRK54* was also shown to induce stress tolerance by activation of abscisic acid and Ca^+^ pathways.^58^

**Table 4.** Gene related to fatty acid biosynthesis and regulation by stress factors.

Gene	Plant material	Stress signaling pathway gene	Stress	Ref.
*GmFAD3* (Omega 3 Fatty acid desaturase)	Mutant and over expression lines	Over expression of GmFAD3 increased the expression GmWRK54	Salinity and drought tolerance increased due to higher osmoprotectants, cooler canopy chlorophyll contents	^53^
*FAD2, FAD6*	Over expression in E. coli	-	Regulate in response to temperature, light and wounding in olives	^54^
*FAD2, FAD6, FAD3*	Differential expression analyses in soybean	Down regulated by ABA stress signaling	Gene expression decreased by 58- 80% due to reproductive water stress	^55^
*FvC5SD C5-sterol desaturase*	Over expression in transgenic soybean line	Up regulated stress signaling pathway	Low accumulation of ROS, and higher activity of enzymes	^56^
*ABSCISIC ACID INSENSITIVE 3* (*GmABI3*)	Over expression transgenic lines in atbi3 mutant lines	*GmABI3* may regulate stress tolerance (heat and cold) in response to ABA and jasmonic acid	*GmABI3* is responsible for seed-specific TAG (6.5%) and long-chain fatty acid (eicosenoic acid increased 7.5%) biosynthesis soybean	^57^
*GmWIN1–5*	Over expression lines	Upregulation of genes encoding phospholipase D (*PLD*), biotin carboxyl carrier protein 1 (*BCCP1*), lysophosphatidic acid acyltransferase 5 (*LPAT5*), and diacylglycerol acyltransferase 2 (*DGAT2*)	Higher concentration of oil and phospho- lipid involved in cell membrane formation and lower leakage under stress	^59^
*ThIPK2* (an inositol polyphosphate kinase from *Thellungiella halophila)*	Over expression transgenic lines		Gene displayed water deficit-, salt- and oxidative-tolerance; an increase of oleic acid (C18:1); and seed size	^60^
*BnFADs*	Expression analyses of 68 *FAD* genes in response to abiotic stress	Differentially regulated in response to cadmium and salinity stress	Expression of eight *FAD* genes increased in cadmium and salinity stress in tolerant accessions only	^61^

## High Oleic Acid Genetic Stocks

Many edible oil crops such as sunflower, soybean and cotton had high concentration of the polyunsaturated fat linoleic acid (ω = 6). Linoleic acid was suitable as salad dresser and handful for bakery products but had poor cooking quality due to low oxidative stability at high temperature. Moreover, such oils had high rancidity and poor shelf life. On the other hand, oils rich in saturated fats had better oxidative and thermo-sensitivity but were considered unhealthy as saturated fat induced cardiovascular diseases.

Oleic acid is a mono-unsaturated fatty acid (ω = 9) which had higher stability under oxidative and thermal stress.^1^ Therefore, development of high oleic acid stock has been of interest in a range of species (Table 5). Genetic stock had high oleic acid concentration (80%) when gene and its homologues *FAD2* (coding enzyme *fatty acid desaturase 2*) were inactivated through a guided protein (sgRNA) (Fig. 3). Guided proteins induced mutation in the reading frame of the gene and its variants in polyploid species.

**Table 5.** High oleic acid mutant stock throug CRISPR/Cs9 in various oilseed crop.

Species (crop)	Gene	Effect	References
Camelina sativa	*FAD2* (*Fatty acid desaturase 2*)	pRedU6fad2EcCas9 vector *DsRed* selection marker, U6 promoter, single guide RNA (sgRNA) covered common region in three *CsFAD2* homoeologs. Knockout posed undesirable effects on plant phenotype. Oleic acid increased (80%). Two pair transformation increased oleic acid (60%) with normal phenotypes	^62^
	Three homologues of FAD2	High oleic acid lines were obtained in T3 and T4 generation	^63^
Gossypium hirsutum (cotton)	*FAD2* mediating ω‐6 fatty acid desaturase	Down regulation of gene increased oleic acid by 80%.	^64^
Thlaspi arvense (pennycress)	*FAD2*, *ROD* (*reduced oleate desaturase*)	*Fad2* or *fad2 fae1* could double oleic acid contents	^65^
Arachis hypogea (peanut)	*FAD2*	Seed-specific down regulation of gene led to increased oleic acid content	^66^
Oryza sativa (rice)	*OsFAD2–1*	Expression of Os FAD2–1 was disrupted which doubled oleic acid contents	^67^
Brassica napus (rapeseed)	fad2_Aa	Increase in high oleic acid contents	^68^
	BnaFAD2	Oleic acid contents increased from 65% to 80%. Mutation in *BnaFAD2* in A5 genome caused more significant fatty acid profile changes	^69^
	BnFAD2 and BnFAE1	Increase oleic acid (70–80%) with decreased erucic acid	^70^
	*BnFAD2*	Poor agronomic performance but high oleic acid content while oil content remain constant	^71^
Glycine max (soybean)	*GmFAD2*	Oleic acid 80% with corresponding decrease in linoleic acid to 1.3%	^72^
		Oleic acid content increased 11% to 40–50% in fad2-1a and fad2-1b mutants, and to 85% in the fad2-1a/fad2	^73^
Arachis hypogea (peanut)	*ahFAD2A*	High oleic acid content	^74^
Elaeis guineensis (oil palm)	*EgFAD2* and *EgPAT*	High oleic acid and stearic acid palm oil	^75^

### Development of Genetic Stock with Low Anti-Nutritional Components

CRISPR/Cas9 gene editing may be potentially used to reduce phytic acid, which is a bind form of phosphorous in seed meal and may not be available to animals and poultry (Table 6). Inactivation of genes such as *BnITPK* and its functional paralogs may increase the free available phosphorous in seed meals. One of the problems with seed meal was the presence of allergens, which may be reduced by knock down of gene *BrajI* and its four homologues in *Brassica napus*.^76^ Gene editing of *FAH12* may reduce the ricinoleic acid in castor bean. which is a toxic component of its oil.^78^

**Table 6.** CRISPR/Cas9 for reduction of anti-nutritional components in oilseed crops.

Species (crop)	Gene	Effect	References
Brassica napus(rapeseed) (rapeseed)	*BnITPK*	Three functional paralogs of *BnITPK* reduced the phytic acid and increase the free available phosphorous	^23^
	BrajI	Four homologues were mutated to reduce the expression of allergen related protein	^76^
	VTE4	Inactivation may lead to the enhancement of γ or δ tocopherol	^77^
Ricinus communis (castor bean)	*FAH12*	Down regulation of *fah12* may result in reduction ricnoleic acid with corresponding increase in oleic acid	^78^
Camelina sativa	*FAE1*	Inactivation of the FAE1 pathway resulted in several fold increase of EPA and DHA in transgenic plant carrying *DHA2015.1* gene	^30^
Glycine max (soybean)	*AIP2a* or *AIP2b*	Increased the protein contents without sacrificing the oil contents. *AIP2* regulate the expression of *ABI3* transcriptional factor	^79^
Camelina sativa	*CsCRUC*	Modification of amino acid with higher proportion of leucine, cysteine and proline	^80^

Tocopherol is a functional molecule in edible and as anti-oxidant in body and help to decrease rancidity of oil. There are several isoforms such as α, β, γ or δ with varying capacity to act as antioxidant. In activation of genes could reduce the conversion of γ, δ to less active forms such as α or β, which were present in higher concentration in seed than other forms.^1^ The gene *VTE4* was noticed to be key in a metabolic pathway that catalyzed the conversion of γ or δ tocopherol to α or β tocopherol in *Brassica napus*.^77^

## Conclusion

Benefits of CRISPR/Cas9 gene editing are obvious and this gene technology may be further utilized for the development of mutant stocks enriched with oleic acid content and with a reduction of anti-nutritional components, or for knocking down genes related with seed physiological traits such as polysaccharide content that enhanced seed oil content. However, the use of this technology is limited in few species, threatened by gene redundancy in polyploidy species, and may also induce off target mutations. In order to fully expand the potential of this technology, new mutant stocks with high stearic contents and modification of tocopherols and sterols contents may be developed in a wide range of oil species to improve the market value of oilseed products.
